# DNA methylation-based subtypes of acute myeloid leukemia with distinct prognosis and clinical features

**DOI:** 10.1007/s10238-022-00980-4

**Published:** 2023-01-16

**Authors:** Jimo Jian, Chenglu Yuan, Chunyan Ji, Hongyuan Hao, Fei Lu

**Affiliations:** 1 Department of Hematology, Qilu Hospital of Shandong University, Qingdao, 266035 Shandong People’s Republic of China; 2 Department of Hematology, Qilu Hospital of Shandong University, Jinan, 250012 Shandong People’s Republic of China

**Keywords:** Acute myeloid leukemia (AML), DNA methylation, Bioinformatics, Immune checkpoint, Prognosis

## Abstract

Acute myeloid leukemia (AML) is a malignancy of the stem cell precursors of the myeloid lineage. DNA methylation is an important DNA modification that regulates gene expression. Investigating AML heterogeneity based on DNA methylation could be clinically informative for improving clinical diagnosis and prognosis. The AML subtypes based on DNA methylation were identified by unsupervised consensus clustering. The association of these subtypes with gene mutation, copy number variations, immune infiltration and clinical features were further explored. Finally, univariate, LASSO and multivariate cox regression analyses were used to identify prognosis-associated genes and construct risk model for AML patients. In addition, we validated this model by using other datasets and explored the involved biological functions and pathways of its related genes. Three CpG island methylator phenotypes (CIMP-H, CIMP-M and CIMP-L) were identified using the 91 differential CpG sites. Overall survival, morphology, macrophages M0 and monocytes were distinct from each other. The most frequently mutated gene in CIMP-L was DNMT3A while which in CIMP-M that was RUNX1. In addition, the TIDE scores, used to predict the response to immune checkpoint inhibitors, were significantly different among CIMPs. The CIMP-associated prognosis risk model (CPM) using 32 key genes had convinced accuracy of prediction to forecast 0.5-year, 1-year, 3-year and 5-year survival rates. Moreover, the risk score-related genes were significantly enriched in pattern specification process, regionalization, embryonic organ morphogenesis and other critical cancer-related biological functions. We systematically and comprehensively analyzed the DNA methylation in AML. The risk model we constructed is an independent predictor of overall survival in AML and could be used as prognostic factor for AML treatment.

**Supplementary Information:**

The online version contains supplementary material available at 10.1007/s10238-022-00980-4.

## Introduction

Acute myeloid leukemia (AML) is a malignancy of the stem cell precursors of the myeloid lineage [1]. It is featured by abnormal immature blast cells expansion, which leads to bone marrow failure and ineffective erythropoiesis [2–4]. Symptoms of AML include anemia, bleeding, infection, fever, and enlargement of liver, spleen and lymph nodes, and bone pain [1]. A numerous studies have shown that radiation, chemical benzene, viruses and genetic factors could induce AML. It is rare in children but most commonly occurs among old people, accounting for about 90% of all leukemia in adults [5].

DNA methylation is one of the most common epigenetic modifications. It is a biological process by which methyl groups are added to the DNA molecule. Methylation can alter the activity of a DNA segment without changing the sequence. Typically, DNA methylation located in a gene promoter acts to repress the gene transcription. DNA methylation has been recognized as an important component of cancer development. In cancer, gene promoter CpG islands acquire abnormal hypermethylation leading to transcriptional silencing [6]. Hypomethylation generally is related to chromosomal instability and loss of imprinting, while hypermethylation is associated with promoters and arise secondary to gene (oncogene/suppressor) silencing. Typically, there is hypermethylation of tumor suppressor genes and hypomethylation of oncogenes [7].

Abnormal methylation in AML is mainly caused by the methylation of CpG islands of key genes, which causes the gene silencing and affects the normal function of cells. Methylation research at gene level provides a new insight for elucidating the occurrence and development of AML. Here, we systematically studied the DNA methylation of AML by integrating TCGA and GEO datasets. We used unsupervised consensus clustering to identify the molecular subtypes of AML based on DNA methylation, and fully studied the association of these subtypes with gene mutation, copy number variations, immune infiltration and clinical features. Finally, we used univariate, LASSO and multivariate cox regression analyses to identify prognosis-associated genes and employed them to construct risk model for AML patients.

## Materials and methods

### Data acquisition

DNA methylation (*n* = 140), RNA-Seq (*n* = 151), Copy Number Variation (*n* = 200), somatic mutation (*n* = 143) and corresponding clinical data (*N* = 697) of LAML patents were downloaded from the Cancer Genome Atlas (TCGA) database [8]. Meanwhile, DNA methylation profiles of blood from healthy volunteers (GSE105420, *n* = 10) were downloaded from GEO database [9]. Gene expression profiles with corresponding clinical data (GSE12417) downloaded from GEO were used as validation dataset [10, 11].

### Preprocess of DNA methylation data from TCGA

The DNA methylation data (CpG sites) were obtained from TCGA according to the following criteria: removal of probes with low quality; removal of probes located in X or Y chromosome; removal of probes associated with single nucleotide polymorphisms (SNPs); and removal probes mapping not uniquely to the human reference genome.

### Identification of prognosis-related DNA methylation sites

The differential DNA methylation sites were identified as follow: the standard deviation (SD) of beta value in AML samples > 0.2 and the mean of beta value in normal samples < 0.05. Kaplan Meier (KM) survival curve and log rank test were further used to screen these differential CpG sites that were significantly related to prognosis (*P* < 0.05).

### Unsupervised clustering of DNA methylation in AML

The prognosis-related DNA methylation sites identified above were then analyzed by unsupervised K-means consensus clustering in ConsensusClusterPlus [12] R package. The best number (K = 3) of consensus clustering was evaluated based on the consensus matrix. K-means clustering of all AML samples was further performed to identify CpG island methylator phenotype (CIMP) of AML.

### Association analysis of CIMP and clinical characteristics

The overall survival (OS) among different CIMP was assessed using KM survival curve and log rank test to investigate the association between CIMP and clinical prognostic in AML. Chi-square or Fisher exact test were used to determine the statistical significance of other clinical features’ distribution (age, gender, etc.) in different CIMP subtypes.

### Gene set enrichment analysis (GSEA)

The gene expression profiles corresponding to the differential DNA methylation sites were first selected. GSEA analysis [13] was then performed by GSEA software using msigdb.v7.0.symbols.gmt gene set [14].

### Association analysis of CIMP with gene mutation, CNV and immune infiltration

The maftools [15] R package was used to retrieve the gene mutations and calculate the tumor mutation burden (TMB) for each AML patient. The GISTIC [16, 17] module in GenePattern [18] was used to predict the significantly CNV regions. Single sample GSEA (ssGSEA) was carried out by GSVA [19] package in R to calculate the infiltration level of 24 different immune cells. TIDE [20] was used to evaluate the response to immune checkpoint inhibitors for all AML patients. Wilcox test and Kruskal–Wallis were used to determine the statistical significance of immune infiltration cell proportion and response to immune checkpoint inhibitors in different CIMP subtypes.

### Construction of CIMP-associated prognosis risk model (CPM)

The differentially expressed genes (DEGs) were firstly identified by Deseq2 [21] with using raw counts calculated by HTSeq [22], based on the CIMP subtypes. Univariate cox regression analysis was then carried out to screen DEGs that were significantly associated with prognosis (*P* < 0.05). The survival-related genes were finally filtered by LASSO regression using glmnet [23] package in R to select key genes. LASSO regression is a popular variable selection method by fitting generalized linear models that reduces variable number by using penalty algorithms to effectively avoid overfitting and finally obtain more widely used models. A risk score was then established and formulated as below:

$$risk\;score = \sum\limits_{i = 1}^{N} {Exp_{i} \times Coe_{i} }$$

where *N* is the number of genes, Exp(i) is the gene expression of each gene, while Coe(i) is the coefficient of LASSO Cox regression. The risk score is equal to the sum of LASSO Cox regression of each gene multiplied by the gene expression levels of each gene. Based on the median risk score, patients were divided into two groups: high- and low-risk groups. KM survival curves were drawn with the R package survminer. Log-rank test was performed to evaluate the statistical significance. The accuracy of risk score to predict 0.5-year, 1-year, 3-year, and 5-year OS rates of AML patients was evaluated using the pROC package in R.P value < 0.05 was considered as statistically significant.

### Validation of CIMP-associated prognosis risk model

GSE12417 dataset with gene expression profiles and corresponding clinical data was used to validate the CIMP-associated prognosis risk model. Other AML-related key genes from previous study were also used to validate our risk model [24].

### Gene ontology and KEGG pathway enrichment analysis

The correlation between risk score and differentially expressed genes was calculated by Pearson correlation analysis. The genes with correlation coefficient > 0.5 and *P* value < 0.05 were regarded as the risk model-related genes. These genes were further used for Gene ontology [25, 26] and Kyoto Encyclopedia of Genes and Genomes (KEGG) [27–30] pathway enrichment analyses, with using clusterProfiler [31] package in R.

### Nomogram construction based on risk score and clinical characteristics

Univariate and multivariable cox regression were used to identify the prognostic-related features in clinical. The nomogram using prognostic-related features was built to predict 1-year, 3-year, and 5-year OS rates of AML patients using rms R package. A receiver operating characteristic (ROC) curve and a calibration curve were constructed to evaluate the accuracy of the nomogram.

### Workflow

**Fig. 1:**
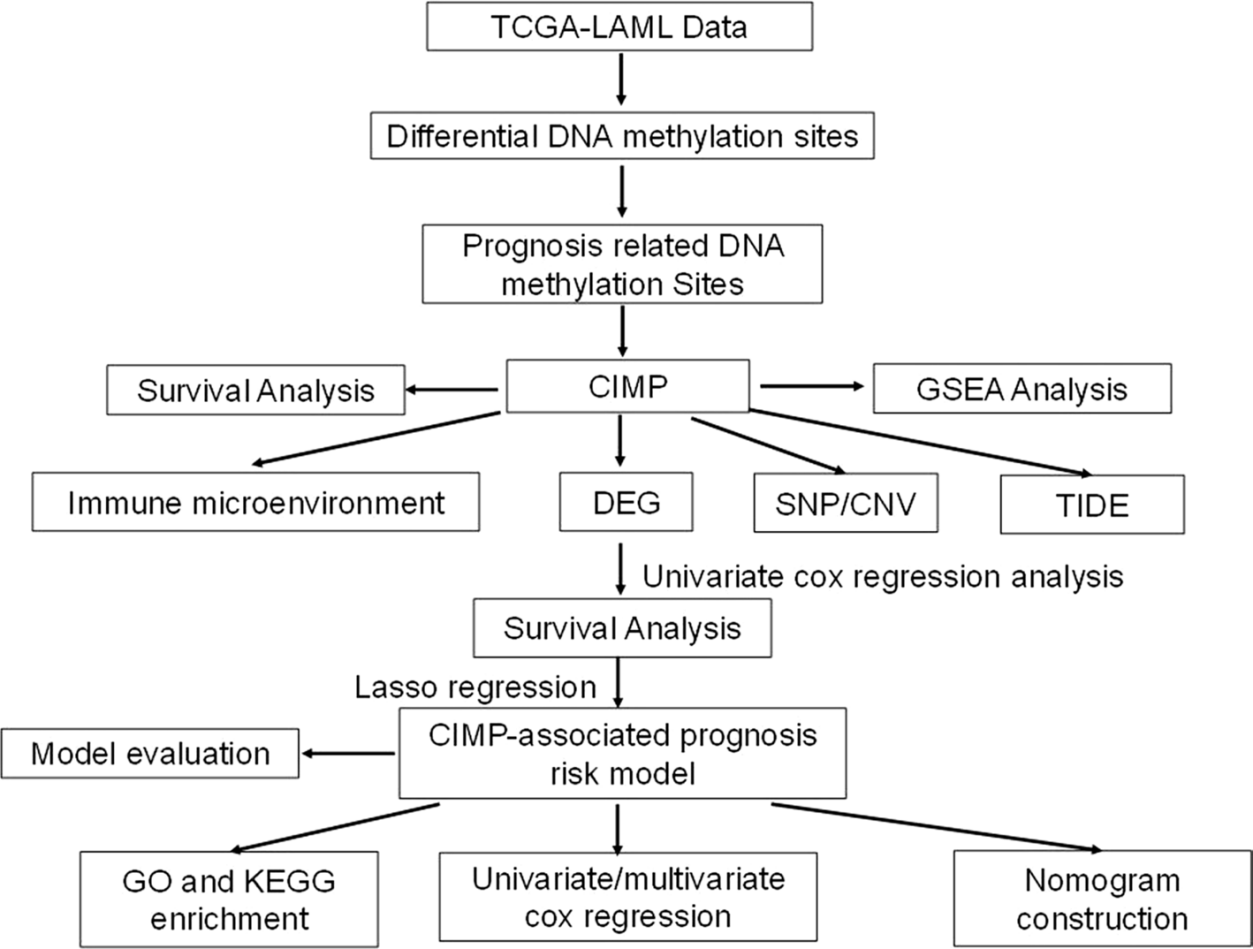
Analysis workflow. TCGA: the Cancer Genome Atlas, *LAML* Acute myeloid leukemia, *CIMP* CpG island methylator phenotype, *GSEA* Gene Set Enrichment Analysis, DEG: differentially expressed gene,*SNP* Single nucleotide polymorphism, *CNV* Copy number variation, *TIDE* tumor immune dysfunction and exclusion, *GO* Gene ontology,*KEGG* Kyoto Encyclopedia of Genes and Genomes

The workflows of the analytical procedures are indicated in Fig. 1 in this study.

## Results

### Prognosis role of DNA methylation in AML

**Fig. 2:**
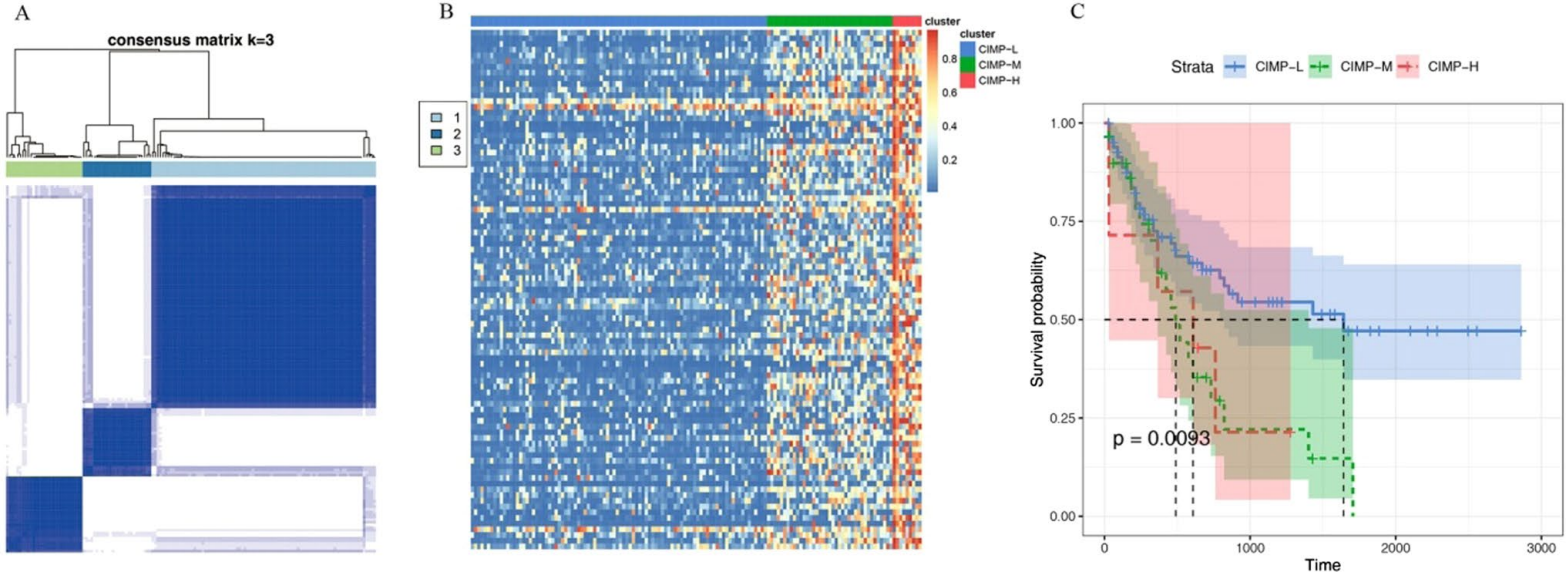
Clustering analysis of DNA methylation in AML samples. **A** Unsupervised consensus clustering of AML samples over prognosis-related DNA methylation sites (K = 3). 1, 2 and 3 represent CIMP-H、CIMP-M and CIMP-L, respectively. **B** Unsupervised K-means clustering of prognosis-related CIMPs in AML. Red and blue indicate upregulated and downregulated DNA methylation level, respectively. **C** Kaplan–Meier OS survival curves of CIMPs (*P* = 0.0093). CIMP-H and CIMP-M associated curves (red and green) are underneath and more sloping than the CIMP-L (blue) associated curve. *CIMP* CpG methylator phenotype, L: low, M: medium, H: high

Developing new prognostic markers to improve therapeutic effects in AML is quite urgent. In this study, we identified differential DNA CpG sites between AML samples (from TCGA) and normal samples (from GES105420). The 91 differential CpG sites, which were significantly related to prognosis (*P* < 0.05), were finally obtained via survival analysis. Then, unsupervised consensus clustering of AML was performed using ConsensusClusterPlus R package. The best K of consensus clustering was 3, based on the consensus matrix (Fig. 2A). Moreover, K-means clustering of all AML samples was performed to identify CpG island methylator phenotype (CIMP). Three CIMPs (CIMP-H、CIMP-M and CIMP-L) were obtained, which were corresponding to high, medium and low DNA methylation levels, respectively (Fig. 2B).

We next investigated the overall survival (OS) among the 3 CIMPs and found CIMP-L and CIMP-H had the best and worst clinical outcomes, respectively (Fig. 2C), indicating DNA methylation sties as excellent prognosis of AML. Moreover, we explored the distribution of clinical features in different CIMP subtypes, including age, gender, morphology and race. Only morphology was found to be significantly associated with CIMP subtypes (Table 1, *P* < 0.05).

**Table 1:** Clinical characteristics of AML samples in different CIMPs

Characteristics	Overall cohort(N = 57)			*P*
	CIMP-L	CIMP-M	CIMP-H	
Number of patients	(n = 95)	(n = 36)	(n = 9)	
Age (mean)	57.11	48.75	42.11	0.32
Gender(Male)	46	21	6	0.397
Gender(Female)	49	15	3	
*Morphology*				0.02
M0	9	3	2	
M1	23	10	4	
M2	14	14	2	
M3	11	3	0	
M4	22	5	1	
M5	15	0	0	
M6	0	0	0	
M7	0	0	0	
*Race*				0.469
Asian	0	1	0	
Black or African-American	8	2	0	
White	86	33	8	

### Gene set enrichment analysis (GSEA) and CIMP-associated phenotype difference

GSEA analysis results showed that CIMP subtypes were all significantly enriched in DNA binding transcription factor activity, animal organ morphogenesis and other pathways. (Supplementary Figs. 1–3).

**Fig. 3:**
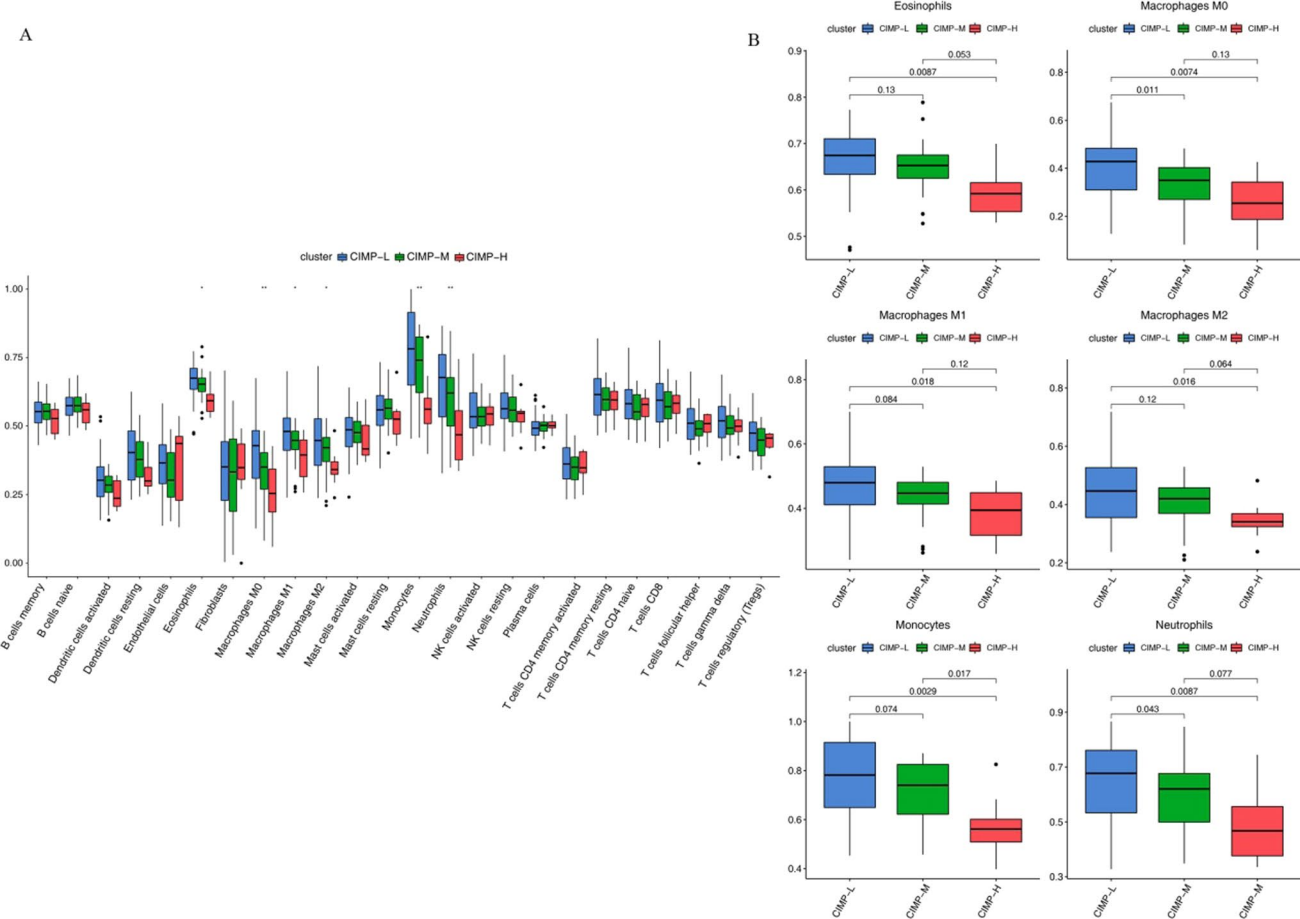
Association between CIMP and immune infiltration levels. The infiltration levels of 24 different immune cells were predicted by ssGSEA. **A** Distribution of 24 immune cells’ proportion among 3 CIMPs. The infiltration levels of eosinophils, macrophages, monocytes and neutrophils were significantly (*P* < 0.05) different among CIMP subtypes as indicted. **B**. Distribution of eosinophils, macrophages, monocytes and neutrophils’ proportion among 3 CIMPs were analyzed by paired comparisons using Wilcox test. *CIMP* CpG methylator phenotype, *ssGSEA* Single sample gene set enrichment analysis

The distribution of different immune cells in three CIMP subtypes is shown in Supplementary Fig. 4. Of those, the infiltration levels of eosinophils, macrophages, monocytes and neutrophils were significantly different among CIMP subtypes (Fig. 3A). Macrophages M0 and monocytes were significantly different among CIMP-L, CIMP-M and CIMP-H comparisons (Fig. 3B).

**Fig. 4:**
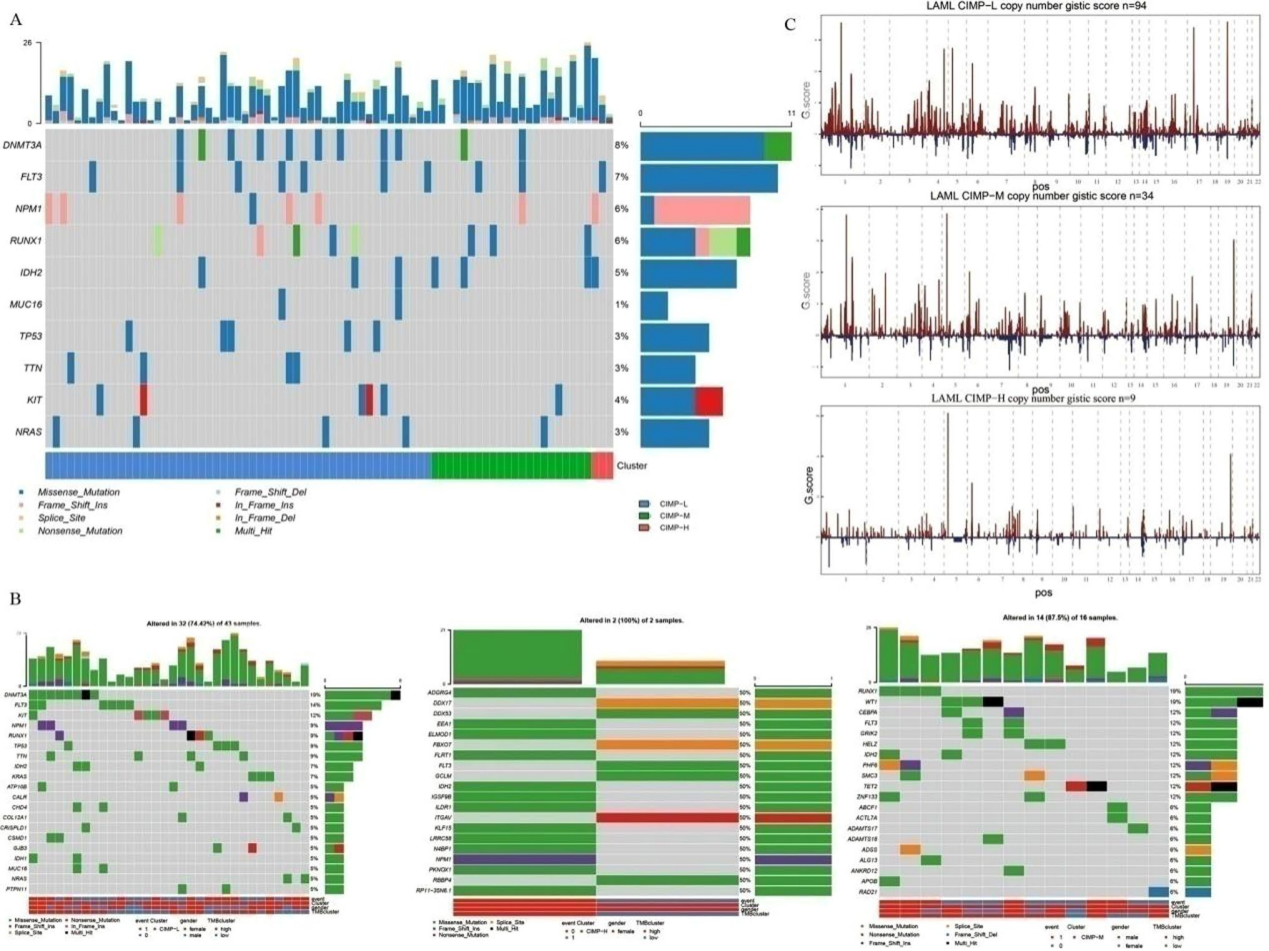
Association between CIMP and genomic alterations. **A**. Mutation profiles of the top 10 mutated genes according to CIMP subtypes. The top and right panel represent the mutation burden and fraction mutation, respectively. Among the 78 patient samples with both gene mutation and DNA methylation information.53, 22 and 3 samples belong to CIMP-L, CIMP-M and CIMP-H, respectively. **B**–**D**. Mutation profiles of the top 20 mutated genes in CIMP-L (**B**), CIMP-M (**C**) and CIMP-H (**D**), respectively. **E** CNV profiles of AML samples with different CIMP subtypes. *CIMP* CpG methylator phenotype

There were 78 patient samples possessing both gene mutation and DNA methylation information. Of them, 53, 22 and 3 patients were assigned to CIMP-L, CIMP-M and CIMP-H subtype groups, respectively. We investigated the top 10 mutated genes according to the CIMP subtypes and found gene *DNMT3A* mutated in 8% of 78 samples, followed by *FLT3* (7%) and *NPM1* (6%) (Fig. 4A).In order to further analyze the specific mutation of the three CIMP subtypes, each subtype was divided into two groups based on the median value of the TMB(Fig. 4B). Surprisingly, we found the most frequently mutated gene in CIMP-L was *DNMT3A,* while *RUNX1* was the most frequently mutated gene in CIMP-M. Moreover, we used GISTIC module in GenePattern to investigate the association of CNV and CIMP subtypes, which is demonstrated in Fig. 4C. There were 78 patients possessing both gene mutation and DNA methylation information. A total of 137 patients were finally obtained, of which 94 were CIMP-L, 34 were CIMP-M and 9 were CIMP-H.

**Fig. 5:**
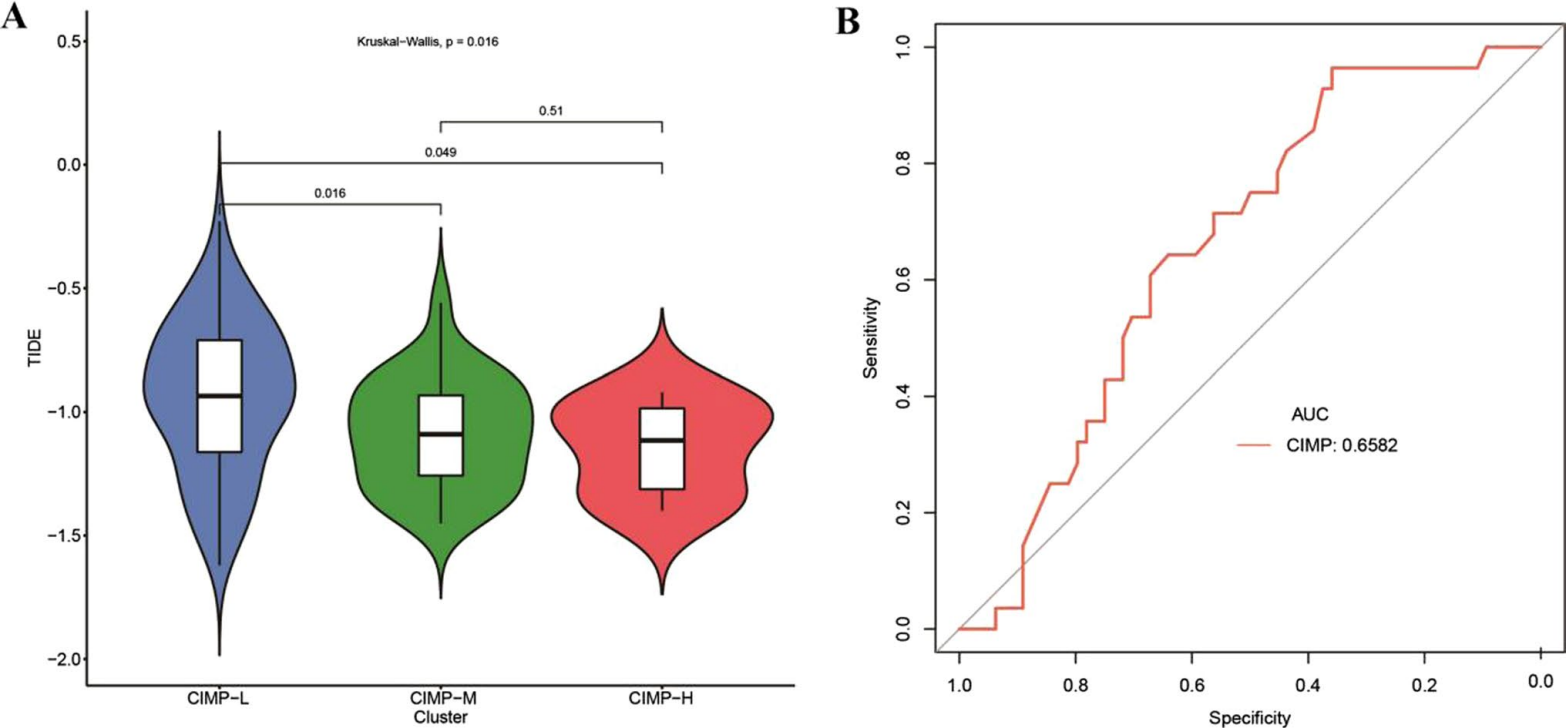
Association between CIMP and response to immune checkpoint inhibitors. **A** Boxplot representation of TIDE scores among different CIMP subtypes. **B** ROC curve of prediction of response to immune checkpoint blockade using CIMP. *CIMP* CpG methylator phenotype, *ROC* Receiver operating characteristic

When predicting the response to immune checkpoint inhibitors, we found TIDE scores were significantly different among different CIMP subtypes (Fig. 5A, *P* = 0.016). In addition, it was found that CIMP subtypes could distinguish well the effect of immune checkpoint treatment via using pROC R package (AUC = 0.6582, Fig. 5B).

### Construction of CIMP-associated prognosis risk model (CPM)

We identified 32 key genes and used them to construct the CIMP-associated prognosis risk model. Briefly, the risk score is equal to the sum of LASSO regression coefficient multiplied by the gene expression levels of each gene (Table 2).

**Table 2:** LASSO regression coefficients of the 32 key genes

Gene	Weight
ITGAE	− 0.00052699
KIF3B	− 0.000494926
C16orf62	− 0.000384884
KIAA0368	− 6.23E−05
BATF2	− 0.003172611
SMARCC1	− 0.000210085
MTX3	− 0.001242344
OR2G3	− 0.003942377
OR2G2	− 0.019387764
C9orf171	0.056999626
SNORA38B	0.000693095
RNU6.1047P	0.058138439
LINC00963	4.24E−05
SLC6A10P	0.542803644
AC104133.1	1.124154419
RNU2.70P	0.01046731
AC063976.3	0.923615203
CFLAR.AS1	0.000320873
CYP21A2	0.148458469
RN7SL788P	0.116595975
ETV5	0.000292585
RWDD4P1	0.013474489
CCDC13	− 0.012335101
PCED1B.AS1	− 0.000905873
KLF3P1	0.003269403
MAPT.AS1	0.266968581
RP13.122B23.9	0.019258711
RP11.697E22.3	0.038717546

**Fig. 6:**
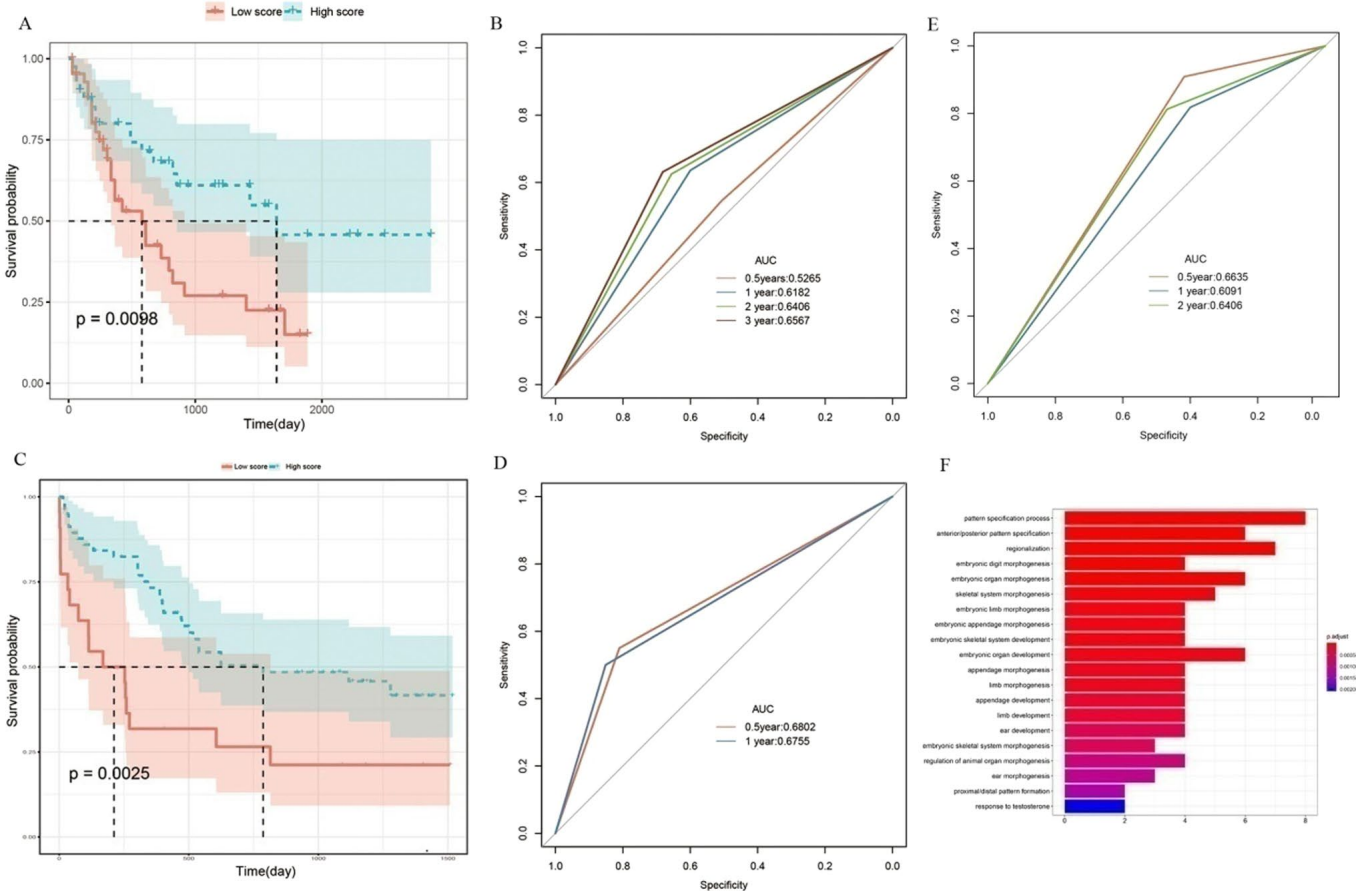
Construction of CIMP-associated prognosis risk model (CPM). Patients were divided into high- and low-risk groups based on the median risk score. **A** Kaplan–Meier OS survival plot of high-risk versus low-risk score groups. **B** ROC curve and AUC values of prediction to forecast 0.5-year, 1-year, 3-year and 5-year survival rates of AML patients using risk score. **C** GO enrichment analysis of risk score-related and differentially expressed genes in AML, x-axis were the − log10 of *p*-value and y-axis was the names of biological processes. *CIMP* CpG methylator phenotype, *AUC* Area under curve, *ROC* Receiver operating characteristic, *GO* Gene ontology

Based on the median risk score, we divided the patients into two groups: high- and low-risk groups. We found the high risk group was significantly related to better survival (Fig. 6A). The AUC values of risk score to predict 0.5-year, 1-year, 2-year, and 3-year OS rates of AML patients were 0.5265, 0.6182, 0.6496 and 0.6567, respectively(Fig. 6B 3-year AUC = 0.6567). Moreover, we validated this finding by using GSE12417 dataset. As expectedly, low risk group was significantly related to poor survival in GSE12417 and the AUC values of survival was larger than 0.67 (Fig. 6C, D).Moreover, we employed other AML-related key genes including NOTCH1, NOTCH2, NOTCH3, NOTCH4, JAGGGED2 and DLL-3, to validate our risk model and got similar results (Fig. 6E, AUC > 0.6).

We found these genes were significantly enriched in pattern specification process, regionalization, embryonic organ morphogenesis and other critical cancer-related biological functions (Fig. 6F).There were no significantly enriched KEGG pathways of these genes.

### Nomogram construction based on risk score and clinical characteristics

**Fig. 7:**
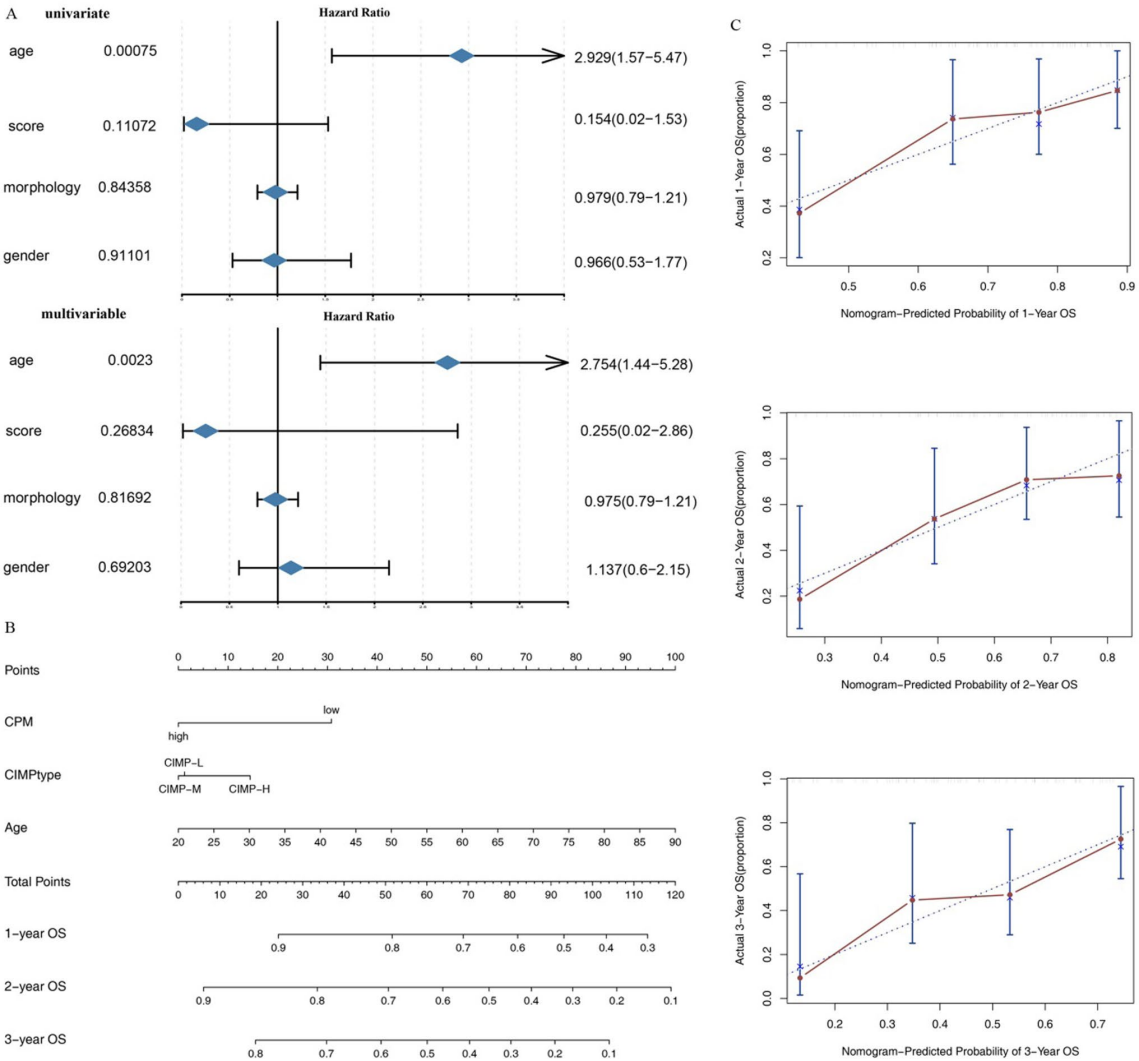
Nomogram for prediction of 1-, 2- and 3-year OSrates of AML patients. **A** Forest plots of age, CPM score, morphology and gender. **B** Nomogram for the prediction of 1-year, 2-year, and 3-year OS rates of AML patients. Find the value on the variable axis and draw a vertical line upward to the ‘Points’ axis and determine the corresponding point for the variable. Add up the points obtained for each variables and locate this sum on the ‘Total Points’ axis. Draw a vertical line down to the 1-, 2- and 3-year OS axis to predict the corresponding OS rates of AML patients. **C** Calibration curves of nomogram-predicted probability of 1-year, 2-year and 3-year survival rates. Vertical bars indicate 95% confidence intervals. *CPM* CIMP-associated prognosis risk model, *CIMP* CpG methylator phenotype, *OS* overall survival

We firstly used univariate and multivariable cox regression to identify the prognostic-related features in clinical, and found age was significantly associated with survival (Fig. 7A). Next we constructed the nomogram consisting of age, CIMP subtype and CPM to predict 1-year, 2-year, and 3-year OS rates of AML patients (Fig. 7B).The results of the nomogram and COX regression analysis were similar. The impact of CPM on the survival rate was small, and basically did not lead to a significant decrease in the survival rate; while CIMP type basically had no impact on the survival rate. However, age was extremely correlated with the survival rate. With the increase in age, the 1-year, 2-year and 3-year survival rates decreased significantly. Calibration curves were applied to evaluate performance of the nomogram, and it was shown that the nomogram had significant accuracy of prediction (Fig. 7C).

## Discussion

AML is a malignant disease of the hematopoietic stem cells’ disorder. Although advancements in therapeutic regimens have been achieved, the 5-year OS is still very poor (about 40%), especially in elderly population older than 60 years old. Therefore, it is quite urgent and necessary to develop new diagnostic and prognostic markers to improve therapeutic effects in AML. DNA methylation plays an important role in cancer occurrence and development by regulating gene expression. Abnormal DNA methylation provide potential biomarkers for the early diagnosis and prognosis of cancer [32–35]. Here, we systematically investigated the DNA methylation and their prognostic roles in AML via integrative analyses by using TCGA and GEO datasets.

There were 91 differential CpG sites in total that were significantly related to prognosis (*P* < 0.05) in AML by integrating TCGA and GES105420 data. Moreover, we identified 3 CpG island methylator phenotypes (CIMP-H、CIMP-M and CIMP-L) through unsupervised consensus clustering and K-means clustering. The survival curves of those CIMPs were distinct from each other. Interestingly, we found morphology was significantly related to CIMP subtypes (*P* < 0.05).All the 3 CIMPs were significantly enriched in DNA binding transcription factor activity and animal organ morphogenesis pathways. Particularly, dysregulated transcription factors regulate abnormal gene expression, including cell death program, differentiation blockade and hallmarks of cancer [36], which represent a unique class of drug targets for future cancer treatment.

By predicting infiltration levels of the 24 immune cells in AML, we discovered macrophages M0 and monocytes were significantly different among CIMP subtypes. Yuan et al. have reported that M0 could increase cancer invasion ability in lung cancer [37]. Moreover, monocytes can influence multiple aspects of cancer development, including anti-tumor immunity, recurrence and metastasis [38]. Elevated tumor-associated monocytes/macrophages could dampen anti-tumor immune response, leading to poor survival [39]. This is coincidently consistent with our findings. What’s more, we found the most frequently mutated gene in CIMP-L was DNMT3A while in CIMP-M that was RUNX1.DNMT3A mutations in AML were associated with poor event free and overall survival [40]. RUNX1 is a relatively infrequent mutational target in AML [41]. Inhibitors of DNMT3Aand RUNX1 could be potentially used for CIMP-Land CIMP-M patients, respectively, in AML to improve their clinical outcomes [42, 43]. In addition, the TIDE scores, used to predict the response to immune checkpoint inhibitors, were significantly different among CIMPs.

Next, we constructed the CIMP-associated prognosis risk model (CPM) using 32 key genes. Impressively, we found the high risk group was significantly related to better survival. The AUC values of risk score to predict 0.5-year, 1-year, 3-year, and 5-year OS rates of AML patients were 0.5265, 0.6182, 0.6496 and 0.6567, respectively. Moreover, we validated this finding by using GSE12417 dataset and other AML-related key genes. These finding indicated the CPM we constructed had significant accuracy of prediction to forecast 0.5-year, 1-year, 3-year and 5-year survival rates. Moreover, we the risk score-related genes were significantly enriched in pattern specification process, regionalization, embryonic organ morphogenesis and other critical cancer-related biological functions. Finally, we built the nomogram consisting of age, CIMP subtype and CPM and the calibration curves demonstrated the nomogram had significant accuracy of prediction of 1-year, 2-year, and 3-year survival rates.

In conclusion, our study provided a systematic and comprehensive view of the DNA methylation in AML. We identified 3 CIMP subtypes based on DNA methylation. Moreover, the CIMP-associated prognosis risk model we constructed using the 33 genes is an independent predictors of OS in AML and could be used as prognostic factor for AML treatment.

### Supplementary Information

Below is the link to the electronic supplementary material.

Supplementary file1 (DOCX 3245 KB)

## Data Availability

The datasets analyzed during the current study are available in the TCGAhttps://portal.gdc.cancer.gov/.

## References

[1] Dohner H, Weisdorf DJ, Bloomfield CD (2015). Acute Myeloid Leukemia. N Engl J Med.

[2] Bain BJ, Bene MC (2019). Morphological and Immunophenotypic Clues to the WHO Categories of Acute Myeloid Leukaemia. Acta Haematol.

[3] Naymagon L, Marcellino B, Mascarenhas J (2019). Eosinophilia in acute myeloid leukemia: overlooked and underexamined. Blood Rev.

[4] Medeiros BC (2019). Optimizing survival outcomes with post-remission therapy in acute myeloid leukemia. Am J Hematol.

[5] Siegel RL, Miller KD, Jemal A (2020). Cancer statistics, 2020. CA Cancer J Clin.

[6] Wang YP, Lei QY (2018). Metabolic recoding of epigenetics in cancer. Cancer Commun (Lond).

[7] Gonzalo S (2010). Epigenetic alterations in aging. J Appl Physiol.

[8] Cancer Genome Atlas Research (2013). Genomic and epigenomic landscapes of adult de novo acute myeloid leukemia. N Engl J Med.

[9] Palomo L (2018). DNA methylation profile in chronic myelomonocytic leukemia associates with distinct clinical, biological and genetic features. Epigenetics.

[10] Wang YH (2021). Distinct clinical and biological characteristics of acute myeloid leukemia with higher expression of long noncoding RNA KIAA0125. Ann Hematol.

[11] Metzeler KH (2008). An 86-probe-set gene-expression signature predicts survival in cytogenetically normal acute myeloid leukemia. Blood.

[12] Wilkerson MD, Hayes DN (2010). ConsensusClusterPlus: a class discovery tool with confidence assessments and item tracking. Bioinformatics.

[13] Subramanian A (2005). Gene set enrichment analysis: a knowledge-based approach for interpreting genome-wide expression profiles. Proc Natl Acad Sci U S A.

[14] Liberzon A (2015). The Molecular Signatures Database (MSigDB) hallmark gene set collection. Cell Syst.

[15] Mayakonda A (2018). Maftools: efficient and comprehensive analysis of somatic variants in cancer. Genome Res.

[16] Mermel CH (2011). GISTIC2.0 facilitates sensitive and confident localization of the targets of focal somatic copy-number alteration in human cancers. Genome Biol.

[17] Beroukhim R (2007). Assessing the significance of chromosomal aberrations in cancer: methodology and application to glioma. Proc Natl Acad Sci U S A.

[18] Reich M (2006). GenePattern 2.0. Nat Genet.

[19] Hanzelmann S, Castelo R, Guinney J (2013). GSVA: gene set variation analysis for microarray and RNA-seq data. BMC Bioinformatics.

[20] Jiang P (2018). Signatures of T cell dysfunction and exclusion predict cancer immunotherapy response. Nat Med.

[21] Love MI, Huber W, Anders S (2014). Moderated estimation of fold change and dispersion for RNA-seq data with DESeq2. Genome Biol.

[22] Anders S, Pyl PT, Huber W (2015). HTSeq–a Python framework to work with high-throughput sequencing data. Bioinformatics.

[23] Friedman J, Hastie T, Tibshirani R (2010). Regularization paths for generalized linear models via coordinate descent. J Stat Softw.

[24] Takam Kamga P (2019). Notch signaling molecules as prognostic biomarkers for acute myeloid leukemia. Cancers (Basel).

[25] The Gene Ontology, C. *The Gene Ontology Resource: 20 years and still going strong.* Nucleic Acids Res, 2019;47(D1):D330-D338. 10.1093/nar/gky1055 PMC6323945 30395331

[26] Ashburner M (2000). Gene ontology: tool for the unification of biology. The Gene Ontology Consortium Nat Genet.

[27] Kanehisa M, Goto S (2000). KEGG: kyoto encyclopedia of genes and genomes. Nucleic Acids Res.

[28] Kanehisa M (2017). KEGG: new perspectives on genomes, pathways, diseases and drugs. Nucleic Acids Res.

[29] Kanehisa M (2019). New approach for understanding genome variations in KEGG. Nucleic Acids Res.

[30] Kanehisa M (2019). Toward understanding the origin and evolution of cellular organisms. Protein Sci.

[31] Yu G (2012). clusterProfiler: an R package for comparing biological themes among gene clusters. OMICS.

[32] Kim DS, Lee WK, Park JY (2018). Promoter methylation of Wrap53alpha, an antisense transcript of p53, is associated with the poor prognosis of patients with non-small cell lung cancer. Oncol Lett.

[33] Li Y (2018). Downregulation of CLDN7 due to promoter hypermethylation is associated with human clear cell renal cell carcinoma progression and poor prognosis. J Exp Clin Cancer Res.

[34] Hao X (2017). DNA methylation markers for diagnosis and prognosis of common cancers. Proc Natl Acad Sci USA.

[35] Ma H (2020). Specific glioblastoma multiforme prognostic-subtype distinctions based on DNA methylation patterns. Cancer Gene Ther.

[36] Bushweller JH (2019). Targeting transcription factors in cancer - from undruggable to reality. Nat Rev Cancer.

[37] Yuan A (2015). Opposite effects of M1 and M2 macrophage subtypes on lung cancer progression. Sci Rep.

[38] Kiss M (2020). Systemic reprogramming of monocytes in cancer. Front Oncol.

[39] Singhal S (2019). Human tumor-associated monocytes/macrophages and their regulation of T cell responses in early-stage lung cancer. Sci Transl Med.

[40] Ley TJ (2010). DNMT3A mutations in acute myeloid leukemia. N Engl J Med.

[41] Jalili M (2018). Prognostic value of RUNX1 mutations in AML: a meta-analysis. Asian Pac J Cancer Prev.

[42] Wong KK, Lawrie CH, Green TM (2019). Oncogenic roles and inhibitors of DNMT1, DNMT3A, and DNMT3B in acute myeloid leukaemia. Biomark Insights.

[43] Mill CP (2019). RUNX1-targeted therapy for AML expressing somatic or germline mutation in RUNX1. Blood.

