# Supplementary material for: DNA methylation-based subtypes of acute myeloid leukemia with distinct prognosis and clinical features
Source: Clin Exp Med. 2023 Jan 16;23(6):2639–49. doi: 10.1007/s10238-022-00980-4 (PMC10543573; doi:10.1007/s10238-022-00980-4)
Supplement: Supplementary file 1 — Supplementary file1 (DOCX 3245 KB) [file 10238_2022_980_MOESM1_ESM.docx]

DNA methylation based subtypes of acute myeloid leukemia with distinct prognosis and clinical features - supplementary figures


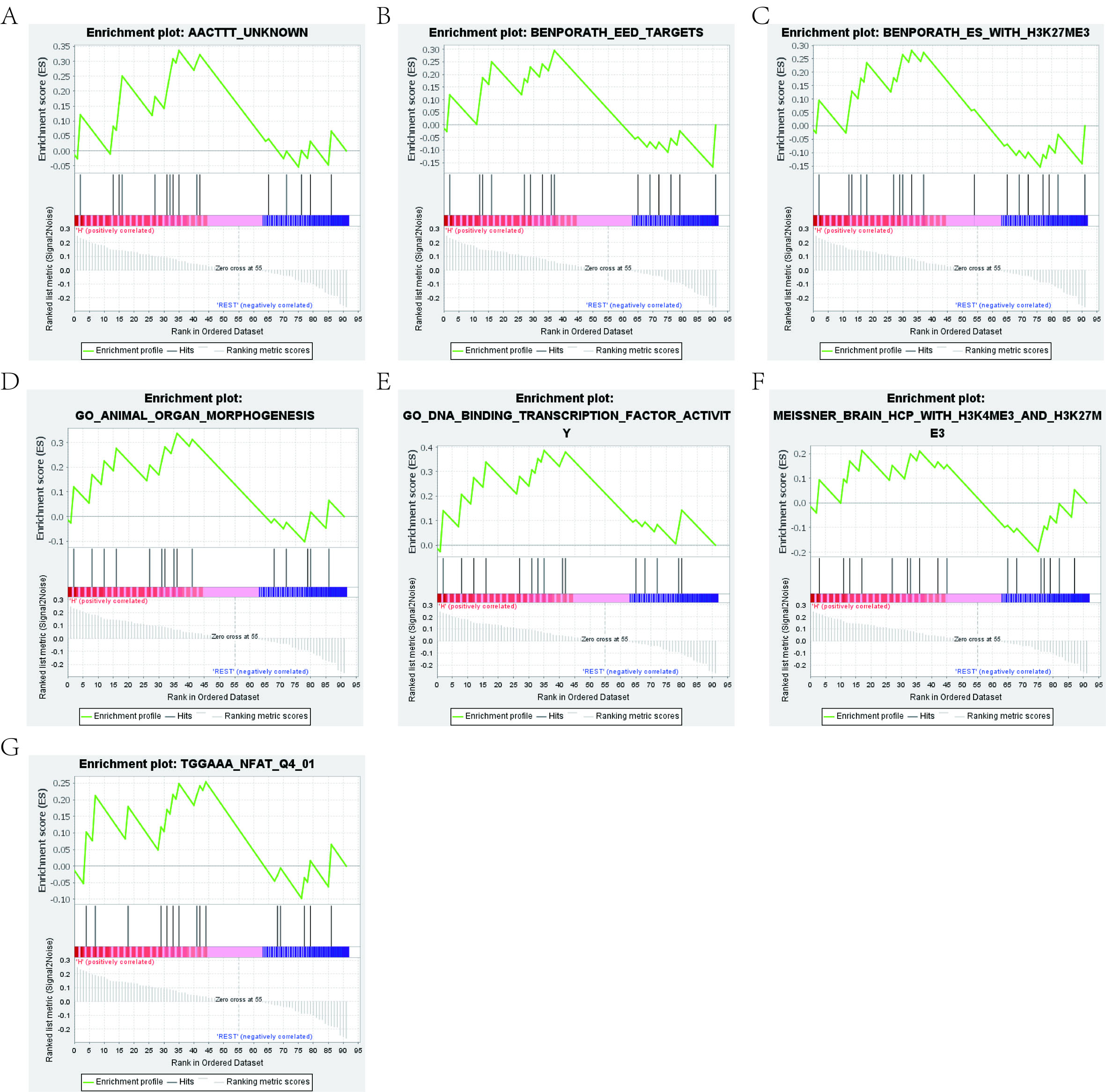


Supplementary figure 1. GSEA enrichment analysis of CIMP-L samples. GSEA: gene set enrichment analysis, CIMP: CpG methylator phenotype.


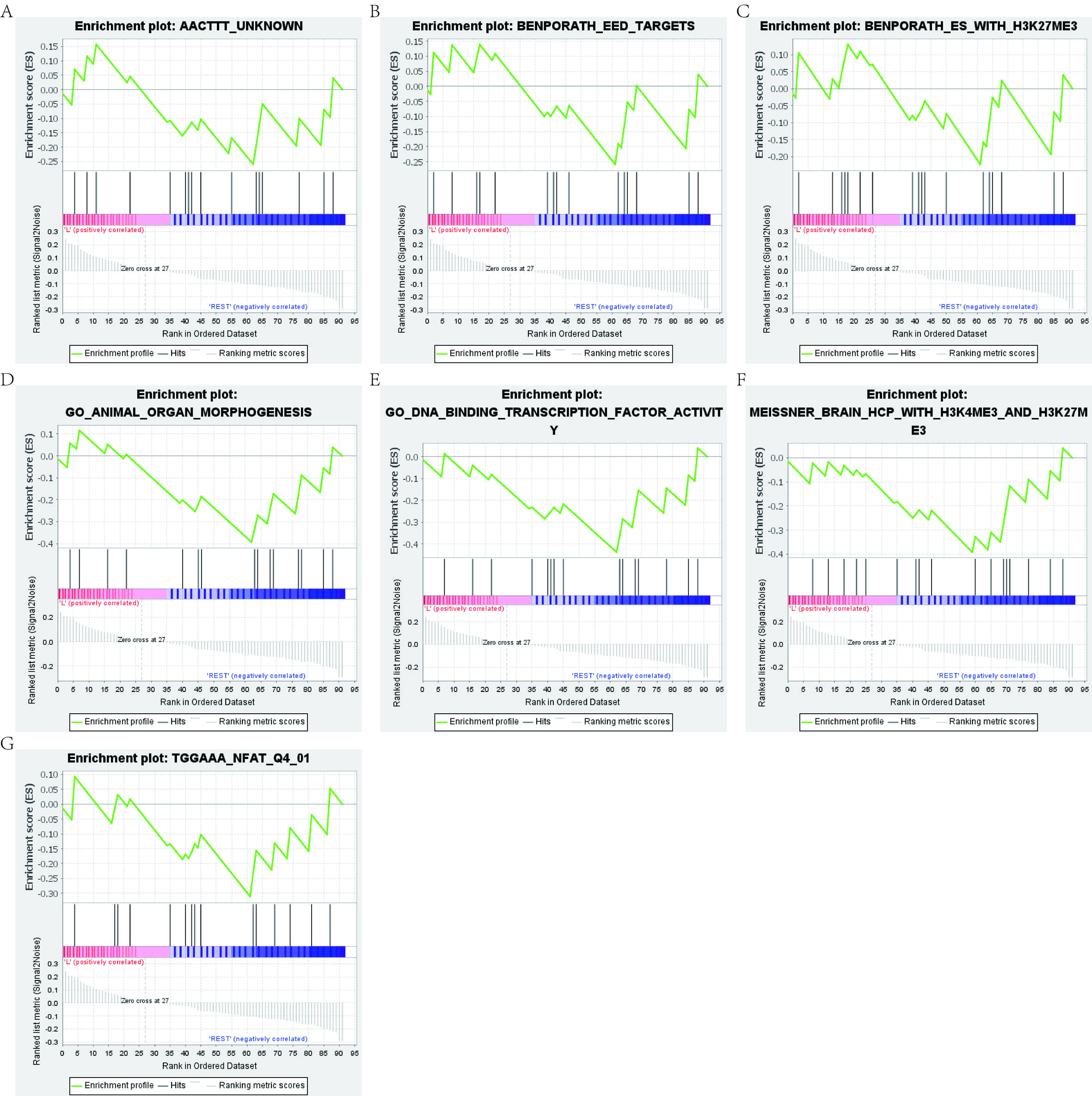


Supplementary figure 2. GSEA enrichment analysis of CIMP-M samples. GSEA: gene set enrichment analysis, CIMP: CpG methylator phenotype.


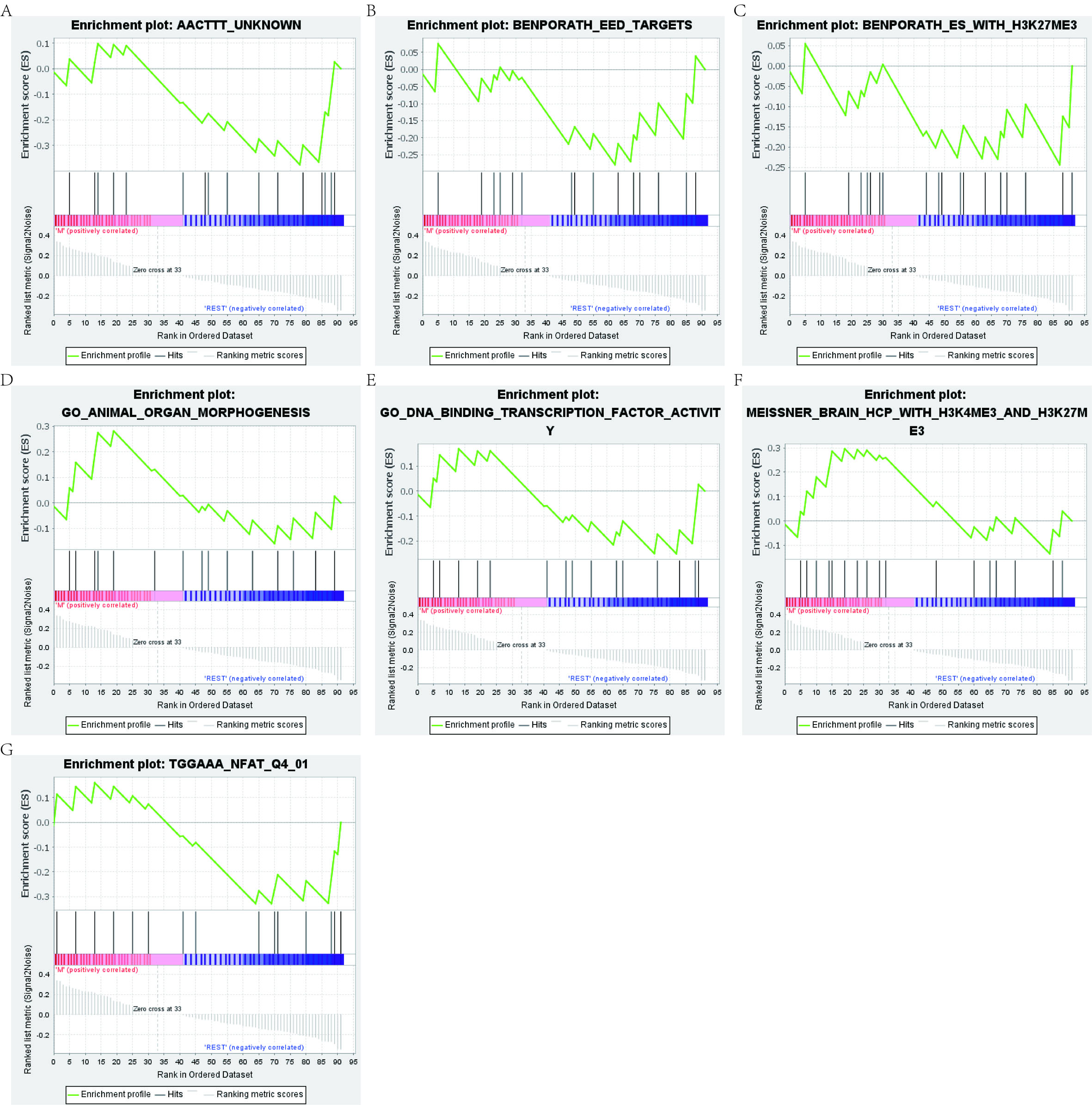


Supplementary figure 3. GSEA enrichment analysis of CIMP-H samples. GSEA: gene set enrichment analysis, CIMP: CpG methylator phenotype.


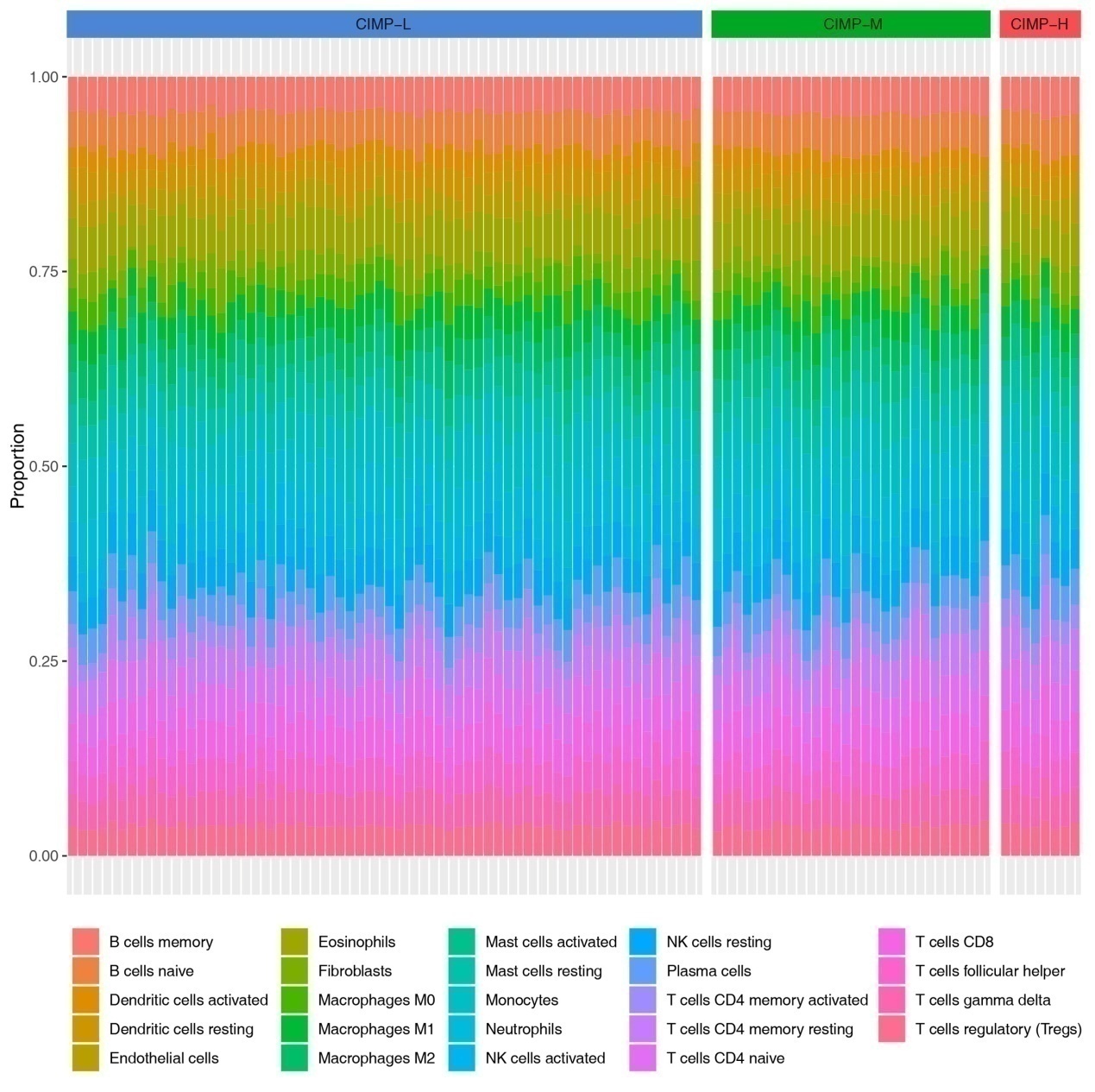


Supplementary figure 4. Immune cell compositions in AML patients with different CIMP subtypes.
